# Imaging NAD(H) Redox Alterations in Cryopreserved Alveolar Macrophages from Ozone-Exposed Mice and the Impact of Nutrient Starvation during Long Lag Times

**DOI:** 10.3390/antiox10050767

**Published:** 2021-05-12

**Authors:** He N. Xu, Joanna Floros, Lin Z. Li, Shaili Amatya

**Affiliations:** 1 Britton Chance Laboratory of Redox Imaging, Department of Radiology, Perelman School of Medicine, University of Pennsylvania, Philadelphia, PA 19104, USA; linli@pennmedicine.upenn.edu; 2 Departments of Pediatrics and Obstetrics and Gynecology, The Pennsylvania State University College of Medicine, Hershey, PA 17033, USA; jfloros@psu.edu; 3 Division of Neonatal-Perinatal Medicine, Center for Host Defense, Inflammation, Lung Diseases (CHILD), Department of Pediatrics, The Pennsylvania State University College of Medicine, Hershey, PA 17033, USA

**Keywords:** bronchoalveolar lavage (BAL), fresh alveolar macrophages, surfactant protein A (SP-A), innate immune responses, oxidative stress, ROS, redox ratio, optical redox imaging

## Abstract

Employing the optical redox imaging technique, we previously identified a significant redox shift of nicotinamide adenine dinucleotide (NAD and the reduced form NADH) in freshly isolated alveolar macrophages (AM) from ozone-exposed mice. The goal here was twofold: (a) to determine the NAD(H) redox shift in cryopreserved AM isolated from ozone-exposed mice and (b) to investigate whether there is a difference in the redox status between cryopreserved and freshly isolated AM. We found: (i) AM from ozone-exposed mice were in a more oxidized redox state compared to that from filtered air (FA)-exposed mice, consistent with the results obtained from freshly isolated mouse AM; (ii) under FA exposure, there was no significant NAD(H) redox difference between fresh AM that had been placed on ice for 2.5 h and cryopreserved AM; however, under ozone exposure, fresh AM were more oxidized than cryopreserved AM; (iii) via the use of nutrient starvation and replenishment and H_2_O_2_-induced oxidative stress of an AM cell line, we showed that this redox difference between cryopreserved and freshly isolated AM is likely the result of the double “hit”, i.e., the ozone-induced oxidative stress plus nutrient starvation that prevented freshly isolated AM from a full recovery after being on ice for a prolonged time period. The cryopreservation technique we developed eliminates/minimizes the effects of oxidative stress and nutrient starvation on cells. This method can be adopted to preserve lung macrophages from animal models or clinical patients for further investigations.

## 1. Introduction

Alveolar macrophages (AM) are the sentinel phagocytic cells of the innate immune system in the lungs [1] and are essential in mediating lung innate immunity and maintaining tissue homeostasis. Lung macrophages are commonly collected through bronchoalveolar lavage (BAL) or tracheal aspiration for subsequent analysis. To minimize the potential impact lag time may have on AM metabolism from the time of macrophage removal from the lung microenvironment to subsequent experimentation, cell ex vivo time should be minimized. Often, BAL samples may not get analyzed immediately after collection (e.g., BAL taken during clinical care) or may need to be shipped to remote sites for analysis. As an alternative, collected lung macrophages can be cryopreserved and revived upon future studies. Although cryopreservation is a common technique for cell culture studies, cryopreserving primary cells can be context-specific and may require fine-tuning of the protocol for better cell revival for subsequent analysis.

Employing the optical redox imaging technique [2,3,4], we have previously investigated the ozone effect on the redox status of coenzyme nicotinamide adenine dinucleotide (NAD and the reduced form NADH, abbreviated to NAD(H)) of freshly isolated AM from humanized surfactant protein A2 (SP-A2) or SP-A knockout (KO) transgenic mice exposed to ozone. The bronchoalveolar lavage fluid was placed on ice and shipped within ~2.5 h to the remote site for imaging analysis. We found ozone exposure shifted NAD(H) to a more oxidized redox status in these freshly isolated AM compared to controls [5]. In the present study we used the same technique to investigate and compare the NAD(H) redox status of cryopreserved AM to that of fresh cells with a lag time on ice prior to experimentation which we previously published [5]. In both cases mice were exposed to ozone or FA prior to AM isolation. Because fresh cells were found to be more oxidized compared to the cryopreserved cells when mice were exposed to ozone, but no such redox difference was observed when mice were exposed to FA, we hypothesized and investigated the following. We postulated that the ~2.5-h time interval during which the fresh cells were kept on ice led to nutrient starvation and that the double “hit”, i.e., ozone exposure and nutrient starvation, placed the fresh cells at a disadvantage for full recovery. Using a mouse alveolar macrophage cell line, we show that this is indeed the case.

## 2. Materials and Methods

### 2.1. Mice, Ozone Exposure, and Macrophage Isolation

All animals used in the present study were male SP-A knockout (KO) mice [6] at 8 weeks of age. The animals were raised and maintained under approved housing in a pathogen-free condition at the Penn State College of Medicine animal facility. The Penn State University College of Medicine Institutional Animal Care and Use Committee approved all procedures involving animals. A total of 12 mice were used, with 7 mice exposed to filtered air (FA) as control and 5 mice exposed to ozone (Table S1). Ozone exposure and macrophage isolation were performed as described in our previous publications [5,7,8,9].

### 2.2. Macrophage Cryopreservation Protocol

The AM from bronchoalveolar lavage were cryopreserved immediately after BAL retrieval according to the following protocol. BAL (~3 mL in volume from adult mice) was retrieved from the anesthetized animal as described above and then centrifuged at 200× *g* for 15 min at 4 °C. After centrifugation, the BAL supernatant was removed carefully without disturbing the cell pellet and stored in −80 °C for future analysis. The BAL cell pellet was then resuspended in 1 mL of the freezing medium and stored in 1.8 mL cryovials. The freezing medium (90% HI-FBS + 10% DMSO) was prepared fresh on the day of the sample collection and chilled to 4 °C prior to use [10]. HI-FBS: heat-inactivated fetal bovine serum was prepared fresh on the day of the experiment by heating fetal bovine serum (Atlanta Biologicals, Flowery Branch, GA, USA) in 56 °C water bath for 30 min. DMSO: dimethyl sulfoxide was purchased from Sigma-Aldrich. The cryovials were then placed in Nalgene Cryo 1 °C freezing containers and then at −80 °C for at least 4 h to achieve a −1 °C/min rate of cooling. Samples were kept at −80 °C until shipment for further experimentation.

### 2.3. Macrophage Preparation for Imaging

The cryopreserved AM stored in −80 °C for less than two weeks were shipped overnight to the destination on dry ice, revived within 2–3 h after arrival and seeded in a glass-bottom dishes, as detailed in our previous report [5].

To simulate the potential effect from shipping AM with ice, we used mouse alveolar macrophage cell line MH-S purchased from ATCC. MH-S cells were routinely cultured in RPMI1640 medium supplemented with 10% fetal bovine serum and 50 µM 2-Mercaptoethanol (MilliporeSigma Company, Burlington, MA, USA) and incubated in 5% CO_2_ and 37 °C according to the ATCC protocol. A total of 100,000 cells in 1 mL medium were seeded in a 4-chamber glass-bottom dish (Cellvis, LLC, Sunnyvale, CA, USA, Catalog # D35C4-20-1.5-N) and incubated for 24 h before starting the starvation experiment.

For the starvation experiments, MH-S cells were rinsed twice with PBS^+^ (PBS with Ca^2+^ and Mg^2+^), followed by adding 0.5 mL of Live Cell Imaging Solution (LCIS, Life Technologies, Carlsbad, CA, USA) spiked with 0 or 135 µM H_2_O_2_. Note that LCIS does not contain nutrients. The dishes were then incubated in 37 °C for 30 min followed by rinsing off H_2_O_2_ with PBS^+^. These dishes were then placed in a 2 °C refrigerator for ~2.5 h followed by imaging. After imaging, the dishes were replaced with LCIS supplemented with 11 mM glucose and 2 mM glutamine for imaging that was performed within 5–20 min.

### 2.4. Optical Redox Imaging and Data Analysis

Approximately 10 min before imaging, the cells were rinsed with PBS^+^ twice, followed by the addition of 1 mL of Live Cell Imaging Solution (LCIS, Life Technologies) supplemented with 11 mM glucose and 2 mM glutamine for imaging.

A Zeiss wide-field microscope (Axio Observer 7, ZEISS, Oberkochen, Germany) set at 37 °C was employed for imaging. Images were acquired with a 20× objective (0.8 NA). NADH signals were collected through optical bandpass filters with excitation (Ex): 370–400 nm and emission (Em): 414–450 nm. Fp (oxidized flavoproteins containing flavin adenine dinucleotide) signals were collected through bandpass filters with Ex: 450–488 nm and Em: 500–530 nm. For imaging mitochondrial reactive oxygen species signals, the MitoSOX^TM^ Red-incubated cells were excited at 385 ± 15 nm and detected at 595 ± 15 nm. Transmitting light was used to locate and focus on regions of interest (ROI) to avoid photo-bleaching. The dishes were first imaged for their redox status followed by 10 min incubation with 2 µM MitoSOX^TM^ Red (Fisher Scientific, Waltham, MA, USA) at 37 °C in the dark. The dishes were rinsed once with PBS^+^, then 1 mL PBS^+^ was added for imaging MitoSOX. Ten randomly selected non-overlapping fields of view per dish were imaged with shading corrections on the fly. The image size was 1920 × 1216 pixels (pixel size 0.29 × 0.29 µm^2^).

Acquired images were processed with a customized routine of Matlab^®^ (MathWorks, Natick, MA, USA). Details of data processing can be found in our previous reports [4,5,11]. Briefly, the cell-free background signals were subtracted from each of the raw images. A signal-to-noise ratio threshold of 7.5 was further applied to exclude the low-intensity pixels. The noise was defined as the standard deviation in the background. The redox ratio Fp/(NADH + Fp) images were generated pixel-by-pixel using NADH, and Fp images were then quantified for the mean values. The net mean values of each of the redox indices (NADH, Fp, and the redox ratio) of each FOV within the same dish were averaged to obtain the dish mean and further averaged across dishes to obtain group means (Table S1).

Statistical analysis was performed with either Student’s *t*-tests assuming unequal variance or ordinary one-way ANOVA tests with post-hoc Tukey’s correction for multiple comparisons using PRISM 9. For each redox index, the results are presented as mean ± standard deviation (SD). *p* < 0.05 was considered statistically significant. Significant differences are displayed as: *, *p* < 0.05; **, *p* < 0.01; ***, *p* < 0.001; and ****, *p* < 0.0001.

## 3. Results

**Figure 1:**
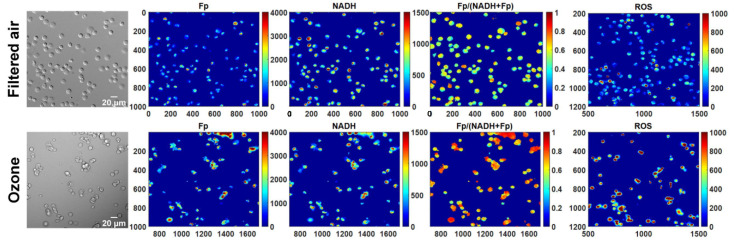
Representative white light and the corresponding redox images of the same field of view for filtered air or ozone exposure, respectively. Remarkable morphological changes can be readily observed in AM from KO mice exposed to ozone, consistent with those observed from freshly isolated AM from ozone-exposed KO mice. The *x* and *y* axes are coordinates of the redox image matrices. Numbers and color bars on the right of each redox image indicate the range of the index being displayed. Note that the ROS images are not in the same field of view as the white light and redox images under FA or ozone exposure, respectively; however, they are from the same animal and cultured in the same dish.

In comparison with freshly isolated AM, the revived AM from cryopreservation appeared to have a similar density of attached cells, indicating no significant cell loss with our cryopreservation procedure. Previously, we observed remarkable morphological changes of fresh AM from KO mice exposed to ozone but AM from FA-exposed KO mice looked normal and healthy [5]. Consistently, as shown in the white light images in Figure 1, ozone exposure resulted in clumpy AM with irregular shapes readily observed in cryopreserved AM after revival. In contrast, AM from FA-exposed mice appeared to be normal and healthy.

**Figure 2:**
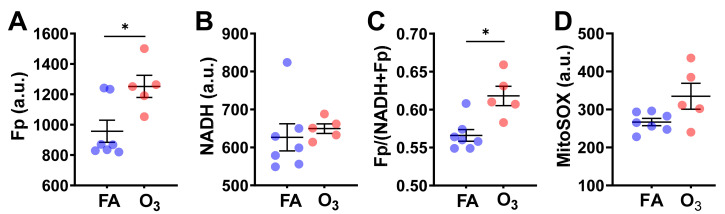
O_3_ induced significant changes in the NAD(H) redox status of cryopreserved AM compared to filtered air (FA). Each dot represents an AM sample from one mouse. (**A**) Significant increase in Fp; (**B**) no significant changes in NADH; (**C**) significant increase in the redox ratio; (**D**) no significant difference in mitochondrial ROS level despite a trend of increase induced by O_3_ (data shown as mean ± SD). * *p* < 0.05.

Quantification of redox imaging data shows a significant NAD(H) redox alteration due to ozone exposure. AM from ozone-exposed mice had an increased Fp value (by 31% increase, *p* = 0.017), no significant change in NADH, and a higher redox ratio (by 9%, *p* = 0.010) (Figure 2A–C), indicating that the cells were in a more oxidized redox state than those of FA exposure. Overall, these results are consistent with our previous findings in fresh AM [5].

**Figure 3:**
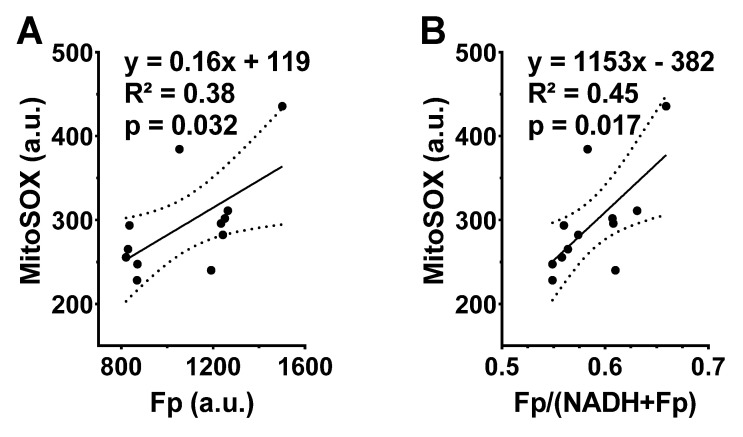
The redox indices Fp and the redox ratio positively correlate with the mitochondrial ROS levels in cryopreserved AM. Each dot represents an AM sample from one mouse, where dashed curves correspond to 95% confidence intervals. (**A**) Higher Fp value significantly correlates with higher ROS level; (**B**) higher redox ratio significantly correlates with higher ROS level.

After imaging the NAD(H) redox status, we subsequently stained the cells with MitoSOX, which allowed us to measure ROS levels in the mitochondria. MitoSOX staining did not detect a significant difference in the level of mitochondrial ROS between the FA-exposed and the ozone-exposed groups despite an uptrend in the ozone group (Figure 2D). This is likely due to the small sample size and large inter-animal variation. Nevertheless, the linear regression analysis shows a significant positive correlation between Fp and ROS level (R^2^ = 0.38, *p* = 0.032) or between the redox ratio and the ROS level (R^2^ = 0.45, *p* = 0.017) (Figure 3), indicating that either higher Fp or higher redox ratio correlates with higher ROS.

**Figure 4:**
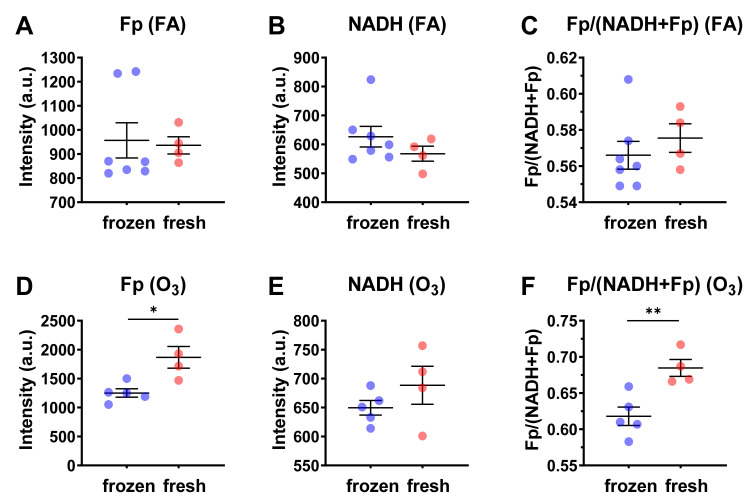
Comparisons of the redox indices between cryopreserved/frozen AM and fresh AM. Each dot represents an AM sample from one mouse. (**A**–**C**) Under FA exposure, there is no significant redox difference between cryopreserved/frozen AM and fresh AM; (**D**–**F**) under O_3_ exposure, fresh AM were in a more oxidized redox state with significantly higher Fp values (**D**) and significantly higher redox ratios (**F**) (mean ± SD). * *p* < 0.05 and ** *p* < 0.01.

Subsequently, we compared the redox indices between revived AM and that of fresh AM under both FA and ozone exposure. The fresh AM in BAL fluid were on ice for ~2.5 h before being cultured in RPMI 1640 medium for imaging. The redox data for the fresh AM were from our previous report [5]. As shown in Figure 4, there is no significant redox difference between revived and fresh AM when the mice were exposed to FA (Figure 4A–C), indicating revived AM and fresh AM had the same redox status. However, ozone exposure did enhance the redox contrast between them. Specifically, the fresh AM had a 49% higher Fp value (*p* = 0.039) and an 11% higher redox ratio (*p* = 0.0060). We suspected that this significant redox contrast was the result of ozone-induced oxidative stress that prevented AM from a full recovery after resuspension and that the fresh AM were at a disadvantage due to nutrient starvation during shipment. The fresh AM were in BAL fluid and stayed on ice for ~2.5 h while being shipped from the animal facility to the site for the imaging analysis. During this shipment, the cells were under nutrient starvation. Upon resuspension in the culture medium, these nutrient-starved cells recovered and re-entered into their original redox status, as shown for those exposed to FA. However, ozone exposure combined with the prolonged (~2.5 h shipping time) nutrient starvation caused significant, possibly permanent cell injuries, thus preventing the cells from a full recovery to the original redox state even upon nutritional replenishment.

**Figure 5:**
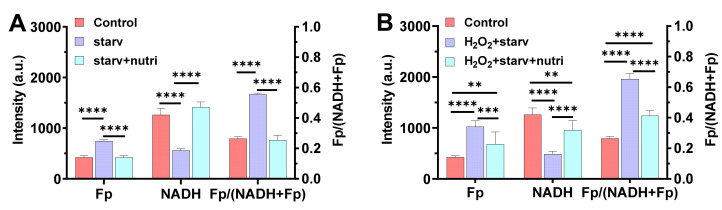
The effect of oxidative stress on the restoration of the NAD(H) redox status of mouse alveolar macrophage cell line MH-S (mean ± SD). (**A**) The NAD(H) redox status was fully restored upon replenishing with glucose and glutamine when AM had no prior oxidative insult before nutrient starvation (*n* = 4), where “starv” = AM in nutrient-free medium at 2 °C for 2.5 h; “starv + nutri” = cells replenished with 11 mM glucose + 2 mM glutamine after “starv”. The results simulate the case of FA exposure (i.e., Figure 4A–C). (**B**) The NAD(H) redox status was only partially restored by replenishing with 11 mM glucose + 2 mM glutamine when AM were first incubated with 135 µM H_2_O_2_ for 30 min at 37 °C prior to nutrient starvation, despite that the added H_2_O_2_ was rinsed off before nutrient starvation (*n* = 5–8). “H_2_O_2_ + starv” = 135 µM H_2_O_2_-pretreated AM underwent nutrient starvation at 2 °C for 2.5 h without the presence of exogenous H_2_O_2_; “starv + nutri” = cells replenished with 11 mM glucose + 2 mM glutamine after “H_2_O_2_ + starv”. The results simulate the case of O_3_ exposure (i.e., Figure 4D–F). ** *p* < 0.01, *** *p* < 0.001, and **** *p* < 0.0001.

To confirm our suspicion, we simulated the nutrient starvation and replenishing processes in a mouse alveolar cell line. The cells were nutrient-starved for 2.5 h in 2 °C. It is known that glucose-starvation results in a more oxidized redox state compared to glucose supplementation [12,13,14]. As expected, we observed that nutrient starvation resulted in a significantly higher Fp (by 75%, *p* < 0.0001), lower NADH (by 56%, *p* < 0.0001), and a higher redox ratio (by 110%, *p* < 0.0001) compared to the control, whereas replenishing the cells with 11 mM glucose and 2 mM glutamine immediately fully restored the NAD(H) redox status (Figure 5A). The three redox indices, Fp, NADH, and the redox ratio, all went back to their original values. In contrast, when the cells were first exposed to 135 µM hydrogen peroxide (H_2_O_2_) for 0.5 h followed by rinsing off the H_2_O_2_, then underwent nutrient starvation for 2.5 h in 2 °C, the same nutritional replenishment only partially restored the redox status despite that the H_2_O_2_ was removed (Figure 5B). Specifically, the hydrogen peroxide-induced oxidative stress plus the nutrient starvation resulted in a 142% Fp increase (*p* < 0.0001), a 67% NADH decrease (*p* < 0.0001), and a 147% increase in the redox ratio (*p* < 0.0001). Apparently, the oxidative stress plus nutrient starvation shifted the cells to a much more oxidized state than nutrient starvation alone. Upon nutrient supplementation, neither Fp nor NADH went back to their original values, and the redox ratio stayed at 0.41 ± 0.034, which is significantly different from the original 0.26 ± 0.013 (*p* < 0.0001). This indicates that exogenous H_2_O_2_-induced oxidative stress profoundly affected an alveolar macrophage’s capacity to regain its redox status upon nutritional replenishment.

## 4. Discussion

Macrophage function is critically supported by cell metabolism, and the essential redox coenzyme pair NAD(H) lies at the heart of this process [15,16]. Oxidative insults, such as ozone and hyperoxia, can adversely affect AM function. In this project, we set out to quantitatively image the NAD(H) redox status in cells that were cryopreserved immediately after collection and then thawed and revived prior to experimentation, as we have done previously using fresh AM [5]. We observed that revived AM had a similar density of attached cells and a similar morphology to fresh AM. This is the first study to our knowledge that investigated cryopreserved mouse alveolar macrophages, as lack of cryopreserved mouse AM analysis has been mentioned as a major limitation in the literature [17]. Nevertheless, our overall observations are consistent with literature reports from other species. For example, using rabbit AM, Lamb et al. reported that no morphologic or adherence characteristics changes occurred after AM were frozen, stored (1 week), and revived; cryopreservation also maintained the primed state of cells [18]. McLemore et al. reported no decrease in human AM viability after 2 weeks of cryopreservation, but a slight decrease in viability after storing in liquid nitrogen for 4 weeks or longer was observed [19]. More recently, Shanthikumar et al. reported 88 vs. 90% live cells between fresh and cryopreserved AM in BAL of three children with cystic fibrosis, using single-cell flowcytometry [20].

We also showed that revived AM overall exhibited a consistent ozone-induced NAD(H) redox oxidative shift with fresh AM from SP-A KO mice exposed to ozone, but to a lesser degree, although no significant difference in their redox indices were found when the mice were exposed to FA. Using a mouse alveolar macrophage cell line, we further investigated why fresh AM had larger redox shifts than the cryopreserved ones by studying the hypothesis that the ~2.5 h lag time of fresh cells on ice prior to experimentation resulted in nutrient starvation. We further hypothesized that this, along with ozone exposure, contributed to a significant difference in redox status between fresh and cryopreserved cells. The results obtained are consistent with this idea. To the best of our knowledge, this is the first study that reports that with prior oxidative stress, fresh mouse AM in BAL fluid placed on ice for ~2.5 h may not be fully recovered to their original metabolic state after resuspension, even if the cells are still viable. The results also indicate that revived AM from cryopreservation could be adopted for future metabolic studies of AM, unless the isolated fresh AM can be immediately placed in culture.

Ideally, the comparison study between cryopreserved and fresh AM should have been performed using AM from the same animals. However, our goal here was to study metabolic changes in AM due to oxidative exposure to animals, by observing relative changes in metabolic state of AM under either cryopreservation or being kept on ice for a prolonged period prior to the next experimental step. Moreover, the use of cryopreserved cells with comparable findings to fresh cells opens up collaborative possibilities among investigators in distant places where each may provide a different set of expertise and/or different facilities/equipment.

Note that AM used in this study were all from male SP-A KO mice. In our previous study using SP-A2 mice, sex was found to play a role in the NAD(H) redox response to ozone [5]. The redox ratio of AM from female SP-A2 mice was more resistant to ozone-induced change than that from males. However, such a sex difference was not significant in AM from SP-A KO mice. Thus, it is likely that cryopreservation will exhibit similar effects on AM from female SP-A KO mice. Studies with additional mouse models are needed to confirm whether sex and SP-A status play a significant role in the beneficial effects of cryopreservation.

## 5. Conclusions

Using the cryopreservation protocol reported here, we found that cryopreserved AM had a similar density of attached cells and morphology to fresh AM from SP-A KO mice. We also found the NAD(H) redox status in cryopreserved AM was shifted to a more oxidized state after mice were exposed to ozone, consistent with the results obtained with fresh AM. However, with prior oxidative stress induced by ozone, fresh AM were more oxidized in their NAD(H) redox status compared to immediately cryopreserved ones. The degree of oxidation difference between the two sets of cells is likely due to the additional oxidative “hit”, i.e., nutrient starvation. This study has major implications for clinical studies where lung macrophages from human tracheal aspirates and BAL may not be possible to be analyzed the same day or at the same location, and thus the cryopreservation of the alveolar macrophages will provide flexibility towards translational bench research. Therefore, the optical redox imaging modality in conjunction with this cryopreservation method may be applied to human lung macrophages to study the alteration of the NAD(H) redox status in patients exposed to oxidative stress.

The present study has both basic science research and clinical impact, and the cryopreservation protocol described can be adopted for the future preservation of lung macrophages from animal models or clinical patients.

## Data Availability

Not applicable.

## Supplementary Materials

The following are available online at https://www.mdpi.com/article/10.3390/antiox10050767/s1, Table S1: Mice used for this study and the redox indices and ROS of their alveolar macrophages.
