# Supplementary material for: Imaging NAD(H) Redox Alterations in Cryopreserved Alveolar Macrophages from Ozone-Exposed Mice and the Impact of Nutrient Starvation during Long Lag Times
Source: Antioxidants (Basel). 2021 May 12;10(5):767. doi: 10.3390/antiox10050767 (PMC8151465; doi:10.3390/antiox10050767)
Supplement: Supplementary file 1 [file antioxidants-10-00767-s001.zip › antioxidants-1202311-supplementary.pdf]

**Supplementary Materials**

**Table S1. Mice used for this study and the redox indices and ROS of their alveolar macrophages**

| Mouse ID | Fp (a.u.) | NADH (a.u.) | Fp/(NADH+Fp) | ROS (a.u.) | Exposure | Age (week) | Sex | SP-A status |
|---|---|---|---|---|---|---|---|---|
| 12341 | 1234 | 650 | 0.608 | 296 | FA | 8 | M | KO |
| 12388 | 868 | 599 | 0.549 | 228 | FA | 8 | M | KO |
| 12389 | 820 | 549 | 0.558 | 256 | FA | 8 | M | KO |
| 12390 | 835 | 579 | 0.560 | 294 | FA | 8 | M | KO |
| 14647 | 829 | 556 | 0.564 | 265 | FA | 8 | M | KO |
| 14648 | 870 | 629 | 0.549 | 247 | FA | 8 | M | KO |
| 14649 | 1242 | 824 | 0.574 | 282 | FA | 8 | M | KO |
| 12339 | 1053 | 614 | 0.583 | 384 | $O_3$ | 8 | M | KO |
| 12340 | 1501 | 651 | 0.659 | 436 | $O_3$ | 8 | M | KO |
| 14591 | 1191 | 633 | 0.610 | 240 | $O_3$ | 8 | M | KO |
| 14592 | 1251 | 688 | 0.607 | 302 | $O_3$ | 8 | M | KO |
| 14645 | 1264 | 662 | 0.631 | 311 | $O_3$ | 8 | M | KO |
